# The path forward: Evolving standards for a smarter digital pathology ecosystem

**DOI:** 10.1016/j.jpi.2025.100516

**Published:** 2025-09-08

**Authors:** Liron Pantanowitz, Anil Parwani

**Affiliations:** University of Pittsburgh, Pittsburgh, PA, USA

## The role of scientific journals

Scientific journals such as the *Journal of Pathology Informatics* (*JPI*) serve as gatekeepers of scholarly communication tasked with disseminating rigorously vetted research. The core responsibilities of JPI include: (1) ensuring scientific integrity through peer review, (2) facilitating discourse by publishing diverse viewpoints, and (3) advancing knowledge by curating impactful, novel findings. This role is not to pick sides but rather to uphold methodological rigor, ethical standards, and editorial transparency. As editors-in-chief, we believe that *JPI* should accordingly publish controversial articles, if the work passes peer review and adheres to ethical and scientific standards.

The recent controversial article published in JPI titled “Wearing a fur coat in the summertime: Should digital pathology redefine medical imaging?” by Peter Gershkovich illustrates this well.^1^ The article critiqued aspects of the DICOM standard, sparking strong reactions. This article appropriately underwent rigorous, blinded peer review. We were fortunate that the two peer reviewers for this article were seasoned informaticians with credible experience in the field of digital pathology. The ensuing consensus reached by *JPI*'s editorial leadership, along with select council members of the Association of Pathology Informatics, was that the blinded peer review process for this article was sound and that the journal's role is to enable exchange of research results and opinions, even if they sometimes challenge prevailing norms. Indeed, the goal of *JPI* is to inform, clarify misconceptions, and invite constructive dialogue—not suppress dissent. Hence, publishing controversial but well-reasoned articles can expose gaps in current understanding, stimulate innovation by challenging assumptions, encourage refinement of existing standards or practices, and promote transparency and accountability in science.

## Summary of the article “Wearing a fur coat in the summertime”

The article by Peter Gershkovich critically examines the limitations of the DICOM standard in digital pathology and advocates for a modular, modern alternative.^1^ The author's central thesis is that DICOM, while foundational in radiology, is architecturally outdated for the needs of digital pathology. Gershkovich points out that DICOM was designed in an era before cloud computing, modular software, and AI-driven workflows. As such, because it embeds metadata, annotations, and communication protocols into a monolithic container, this author's opinion is that DICOM in fact may on occasion limit interoperability, introduce cybersecurity vulnerabilities, as well as restrict scalability and innovation. With modular design as a solution, the author argues that separation of components instead of embedding them into one file allows them to evolve independently, as well as improve performance, security, and adaptability. Further, the article boldly critiques the notion that DICOM guarantees interoperability. Gershkovich notes that DICOM implementations vary widely, often requiring vendor-specific customization. Moreover, he emphasizes that pathology's complex workflows probably demand more than basic image exchange. For example, unlike radiology, he explains that annotations in pathology are more dynamic and complex and that embedding them within image files limits scalability and complicates versioning and access control. This opinion piece published in *JPI* concludes that digital pathology must evolve beyond DICOM's constraints. While DICOM can still play a role, Gershkovich believes it should be reimagined as an interoperability facilitator, not a monolithic container.

## Summary of response letters

As anticipated, the aforementioned article dismayed some *JPI* readers and the DICOM community. David Clunie, who is a radiologist, medical informaticist, DICOM open-source software author and editor of the DICOM standard responded with a critical letter to the editor. Dr. Clunie rejects the claim that DICOM's unified container design limits interoperability or introduces vulnerabilities. He defends DICOM's security capabilities, attributing vulnerabilities instead to poor implementation. In his opinion, DICOM is a comprehensive, secure, and interoperable standard. His letter also emphasizes DICOM's flexibility and modularity, allowing integration with other standards such as FHIR. He thus advocates for evolutionary improvements to DICOM rather than a paradigm shift.

Members of DICOM Working Group 26 (WG-26) also submitted a collaborative and constructive letter to the editor. They clarify and defend DICOM's role in digital pathology and highlight real-world deployments demonstrating successful interoperability. They also emphasize modular, self-contained datasets that enhance resilience, address metadata duplication concerns that affirm DICOM's flexibility, and acknowledge its compatibility with modern encryption and regulatory standards. It is edifying that they encourage community engagement to improve the DICOM standard and promote secure, interoperable digital pathology.

Peter Gershkovich's rebuttal to the WG-26 letter emphasizes the need for a critical re-evaluation of the DICOM standard considering evolving technological and clinical realities. He appreciates the dialogue but maintains that digital pathology is at a unique inflection point requiring scrutiny of inherited standards. Gershkovich reiterates concerns about embedding mutable clinical metadata in image files arguing that it conflicts with NIST's Zero Trust Architecture and compromises data integrity. At Yale, Gershkovich declares that not only did his team successfully integrate digital slides with a custom LIS using modular technologies instead of DICOM but that their implementation was cost-effective and rapidly deployed, contrasting with the complexity and expense he feels may result with a DICOM-based approach. He critiques WG-26's cited success stories as reliant on exceptional institutional resources, not representative of typical pathology departments. He concludes that pathology workflows are specimen- and patient-centric requiring orchestration beyond image management. Gershkovich too calls for broader engagement to ensure standards support diagnostic excellence and patient care.

## Editorial summary: Digital pathology standards debate

Over the course of three published letters, *JPI* has hosted a robust and thoughtful exchange on the future of digital pathology standards—specifically, the role of DICOM in whole slide imaging workflows. Peter Gershkovich initiated this discussion with a critique of DICOM's monolithic architecture arguing that it is misaligned with modern modular, cloud-native, and AI-driven pathology workflows. Subsequent letters to the editor defend DICOM as a mature, flexible, and secure standard and reject the notion that DICOM limits innovation. The authors of these letters dispute the original article's characterization of DICOM as outdated or limiting, emphasize real-world success stories (e.g., Michigan Medicine, Endeavor Health) and practical interoperability (e.g., DICOM/IHE Connectathon), advocate for evolutionary refinement rather than radical replacement, and encourage broader community engagement to strengthen the well-governed DICOM standard.

As editors-in-chief, we commend all these contributors for their rigor, transparency, and commitment to advancing digital pathology. This exchange reflects the vitality of our field and the importance of open, evidence-based discourse. Our recommendation is to continue supporting DICOM as a foundational standard for image exchange, while acknowledging its limitations and encouraging modular enhancements. The goal for all stakeholders is to prioritize interoperability that is functional, secure, and clinically meaningful, not just technically feasible. As such, it remains important to promote inclusivity in standards development by engaging a broader community—including pathologists, informaticians, engineers, cybersecurity experts, industry, and regulators.

In conclusion, *JPI* remains committed to fostering dialogue that bridges theory and practice, and we invite continued contributions that challenge assumptions, share implementation experiences, and propose forward-looking solutions. Let us move forward collaboratively, ensuring that the standards we adopt truly serve the needs of patients, laboratories, and the future of diagnostic medicine.

## Declaration of competing interest

The authors declare the following financial interests/personal relationships which may be considered as potential competing interests:

**Table:** 

Liron Pantanowitz and Anil Parwani report a relationship with the *Journal of Pathology Informatics* and API. Given this role of both authors, their involvement in the peer review of this commentary was limited.
